# Empirical Anatomical Versus HAFE‐Guided Cardioneuroablation for Vasovagal Syncope: Long‐Term Efficacy and Safety Outcomes

**DOI:** 10.1002/clc.70348

**Published:** 2026-05-18

**Authors:** Bo Zhang, Xianfeng Du, Chenxu Luo, Xinzhi Yu, Shenyuan Zhou, Yongxing Jiang, Yin Xu, Renyuan Fang, Mingjun Feng, Caijie Shen

**Affiliations:** ^1^ Health Science Center Ningbo University Ningbo City China; ^2^ Chinaco Healthcare Corporation Limited International Hospital; ^3^ Arrhythmia Center The First Affiliated Hospital of Ningbo University, Ningbo First Hospital Ningbo City China; ^4^ Department of Cardiology, Ningbo No. 2 Hospital Wenzhou Medical University Ningbo City China

**Keywords:** cardioneuroablation, ganglionated plexi, quality of life, radiation exposure, syncope recurrence

## Abstract

**Background and Aims:**

Vasovagal syncope (VVS) poses challenges despite therapy, and optimal cardioneuroablation (CNA) targeting ganglionated plexi (GPs) remains debated. This study compared long‐term efficacy and safety between empirical anatomical‐guided CNA (EAGC) and high‐amplitude fractionated electrogram (HAFE)‐guided CNA in patients with refractory VVS.

**Methods:**

This single‐center retrospective analysis (July 2018 to July 2023) included 109 patients with refractory VVS undergoing CNA. Patients were divided into the EAGC group (*n* = 70; empirical GP ablation at common anatomical sites) or the HAFE group (*n* = 39; HAFE mapping‐guided GP ablation). The primary endpoint was 24‐month syncope‐free survival; secondary endpoints included procedural metrics, quality of life (QoL, via syncope dysfunction score [SDS]), and procedure‐related complications.

**Results:**

No significant difference in 24‐month syncope‐free survival was observed between groups (HR = 1.648, 95% CI 0.648–4.194; log‐rank *p* = 0.285), with rates of 85.6% in the EAGC group and 77.2% in the HAFE group. The HAFE group demonstrated shorter procedure time (median 66.00 min [IQR 52.00–87.50] vs. 90.00 min [IQR 71.75–101.50]; *p* < 0.001) and reduced radiation exposure (X‐ray dose: 6.00 [IQR 4.17–10.75] mGy vs. 12.00 [IQR 6.48–20.00] mGy; *p* = 0.002) and fluoroscopy time (2.53 [IQR 1.92–4.06] min vs. 4.38 [IQR 2.47–7.05] min; *p* < 0.001) compared to those in the EAGC group. Both groups exhibited significant reductions in syncope dysfunction score (SDS; *p* < 0.001), however, the intergroup difference in score reduction failed to reach statistical significance (ΔSDS; *p* = 0.487). Complication rates were comparable between two groups.

**Conclusions:**

EAGC and HAFE‐guided CNA demonstrated comparable 24‑month syncope‑free survival with no significant difference in recurrence risk, while HAFE‐guided CNA improved procedural efficiency.

## Summary

- What's new?
  his study provides the first direct long‐term comparison of empirical anatomical‐guided (EAGC) versus HAFE‐guided cardioneuroablation (CNA) for refractory vasovagal syncope.
  Both EAGC and HAFE‐guided CNA demonstrated comparable 24‐month syncope‐free survival and recurrence risk reduction, with no statistically significant difference between groups.
  HAFE‐guided CNA significantly reduced median procedure time (66.00 vs. 90.00 min) and radiation exposure compared to EAGC, while maintaining comparable safety.

## Graphical abstract

EAGC and HAFE‐guided cardioneuroablation achieve equivalent 24‐month syncope‐free survival and similar quality of life improvements. HAFE‐guided ablation significantly reduces procedure time and radiation exposure while maintaining comparable safety, supporting its procedural efficiency for vasovagal syncope.

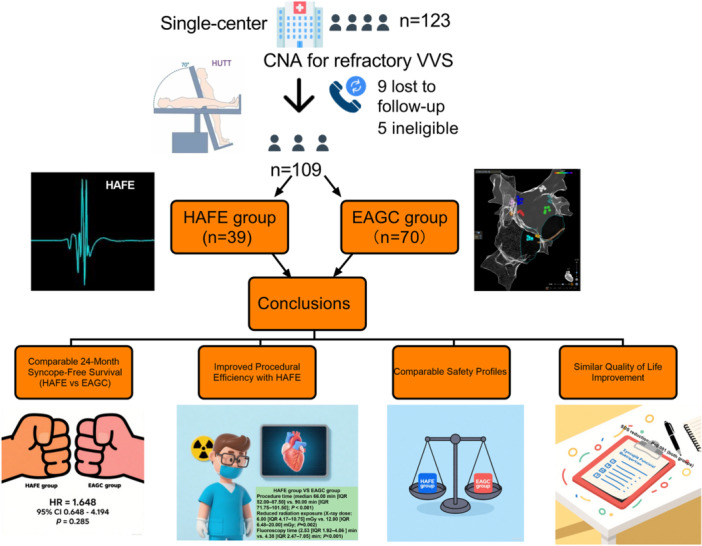

**Abbreviations**
AF: atrial fibrillation
AFN: atrial fibrillation nest
AFN‐HAFE: atrial fibrillation nest‐high‐amplitude fractionated electrogram
CHD: coronary heart disease
CNA: cardioneuroablation
CS: coronary sinus
EAGC: empirical anatomical‐guided cardioneuroablation
GP: ganglionated plexus
HAFE: high‐amplitude fractionated electrogram
HFS: high‐frequency stimulation
HUTT: head‐up tilt test
IVC: inferior vena cava
LA: left atrial
LIGP: left inferior GP
LIPV: left inferior pulmonary vein
LSGP: left superior GP
LSPV: left superior pulmonary vein
LVEDD: left ventricular end‐diastolic diameter
LVEF: left ventricular ejection fraction
LVESD: left ventricular end‐systolic diameter
MA: mitral annulus
MTGP: marshal tract GP
PMLG: posteromedial left atrial GP
QoL: quality of life
RA: right atrium
RIGP: right inferior GP
RIPV: right inferior pulmonary vein
RSGP: right superior GP
RSPV: right superior pulmonary vein
SDS: syncope dysfunction score
SFSQ: Syncope Functional Status Questionnaire
SVC: superior vena cava
SVC‐Ao GP: superior vena cava–aorta GP
T2DM: type 2 diabetes mellitus
TA: tricuspid annulus
VRs: vagal responses
VVS: vasovagal syncope

## 1 Introduction

Cardioneuroablation (CNA) has been validated as an effective therapeutic intervention for patients with refractory vasovagal syncope (VVS), particularly in symptomatic individuals unresponsive to pharmacological therapy [1].

In CNA procedures, identification of ablation targets is critical. Three ablation methods are currently widely utilized in clinical practice: (1) High‐frequency stimulation (HFS)‐guided ablation, which utilizes electrical impulses with a frequency of 20 Hz, amplitude up to 20 V, and pulse duration of 4–5 ms, aids ganglionated plexi (GPs) localization and ablation verification by eliciting vagal responses (VRs). However, this technique carries risks of inducing atrial fibrillation and patients' discomfort (e.g., chest pain), necessitating enhanced sedation and analgesia during the procedure [2, 3, 4, 5]. (2) Empirical anatomical‐guided ablation: ablation is performed in predefined GP regions (e.g., right superior GP [RSGP], right inferior GP [RIGP], left superior GP [LSGP], and left inferior GP [LIGP]), but faces challenges from anatomical variability [6, 7, 8]. (3) High‐amplitude fractionated electrogram (HAFE)‐guided ablation: This approach targets HAFE to localize GPs. Building upon the foundational “AF Nest” concept introduced by Pachon et al. [1, 9], Lellouche et al. [10] further characterized HAFE as a morphological variation; hence, it is also referred to as AFN‐HAFE. By identifying and ablating sites exhibiting HAFE, this technique enables GP localization without the need for additional dedicated devices, thereby reducing procedural and fluoroscopy time [11]. However, its sensitivity for GP identification may be limited [12].

Currently, no consensus exists regarding which intraoperative strategy for ablation target identification minimizes syncope recurrence. Therefore, this study has been designed to directly compare empirical anatomical‐guided CNA (EAGC) with HAFE‐guided CNA in refractory VVS patients, with a focus on evaluating long‐term efficacy and safety outcomes.

## 2 Methods

### 2.1 Study Population

This retrospective study at the Arrhythmia Center of the First Affiliated Hospital of Ningbo University enrolled consecutive patients with refractory VVS undergoing CNA from July 2018 to July 2023.

**Figure 1:**
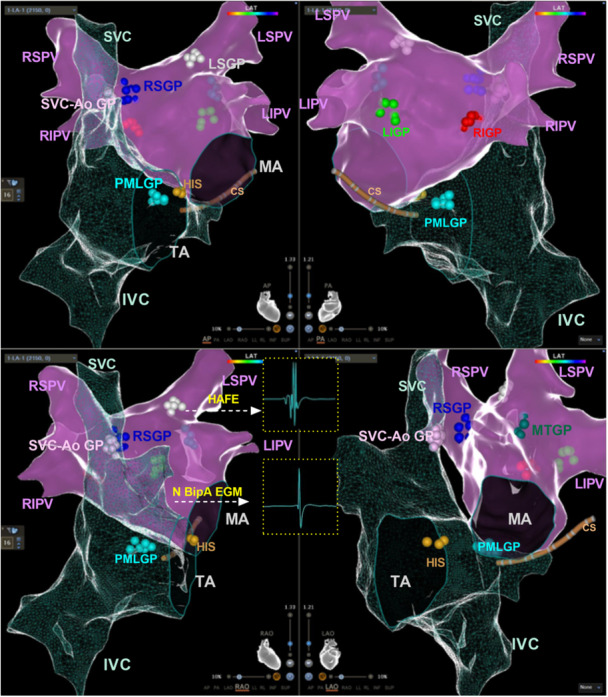
Anatomical schematic of key atrial GPs. CS, coronary sinus; GP, ganglionated plexi; HAFE, high‐amplitude fractionated electrogram; HIS, his bundle; IVC, inferior vena cava; LIGP, left inferior GP; LSGP, left superior GP; LIPV, left inferior pulmonary vein; LSPV, left superior pulmonary vein; MA, mitral annulus; MTGP, marshal tract GP; N BipA EGM, normal bipolar atrial electrogram; PMLG, posteromedial left atrial GP; RIGP, right inferior GP; RIPV, right Inferior pulmonary vein; RSGP, right superior GP; RSPV, right superior pulmonary vein; SVC, superior vena cava; SVC‐Ao GP, superior vena cava–aorta GP; TA, tricuspid annulus.

Eligible participants were selected based on the following inclusion criteria: consistent with the diagnosis of VVS [13], with ≥3 syncopal episodes, and refractory to or intolerant of either previous pharmacological therapy (such as midodrine or β‐blockers) or non‐pharmacological therapy (such as physical counterpressure maneuvers); positive head‐up tilt test (HUTT) [13, 14]; The exclusion criteria were as follows: psychiatric disorders or cognitive impairment; secondary syncope etiologies (e.g., aortic stenosis, ventricular arrhythmias, drug‐induced syncope); structural heart disease (hypertrophic cardiomyopathy, pulmonary hypertension); neurological comorbidities (epilepsy, prior stroke, transient ischemic attack); acute cardiac events (myocardial infarction ≤ 6 weeks prior); severe heart failure (New York Heart Association [NYHA] class III–IV), prior cardiac surgery, or left atrial (LA) thrombi. The study population was stratified into two groups: the EAGC group and the HAFE group. From 2018 to 2020, EAGC was the predominant procedure, and patients treated during this period were enrolled in the EAGC group. From 2021 to 2023, as the HAFE mapping technique matured at our center, HAFE‐guided CNA was progressively adopted as the primary approach, and enrollment of patients in the HAFE group began accordingly. Both groups adhered strictly to the same inclusion and exclusion criteria described above. The study process and patient enrollment are depicted in Supporting Information S1: Figure 1.

All patients were provided written informed consent. In line with the Declaration of Helsinki's ethical principles, this study received approval from the hospital's Ethics Committee.

### 2.2 Intraoperative Mapping and Endpoints in GP Ablation

Preoperative routine preparations, including cardiac echocardiography and fasting was required on the day of the procedure. Radiofrequency ablation (RFA) was performed under deep sedation and local anesthesia. Following femoral venous access, intravenous heparin was administered to maintain an activated clotting time between 250 and 350 s during the procedure. A steerable decapolar catheter was advanced into the coronary sinus vein for intracardiac electrogram recording and to serve as an anatomical reference during transseptal puncture. A quadripolar catheter was positioned at the right ventricular apex to provide ventricular pacing backup in cases of severe intraoperative bradycardia or transient asystole. Intracardiac electrograms were recorded using a multichannel electrophysiology system (EP‐Workmate, Abbott, USA). Bi‐atrial geometric reconstruction and mapping were performed using a multipolar mapping catheter (Pentaray Nav, Biosense Webster, USA) under the guidance of the three‐dimensional electroanatomic mapping (EAM) system (CARTO 3, Biosense Webster, USA). RFA was conducted with an open‐irrigated contact force (CF)‐sensing catheter (Thermocool SmartTouch, Biosense Webster, USA), targeting CFs of 5−20 g, with an irrigation flow rate of 18−30 mL/min. Ablation was performed in power‐controlled mode, delivering radiofrequency energy at a power range of 30–40 W (35 W most frequently used). Lesions were guided by quantitative metrics (ablation index: 500−550 for the anterior LA wall; 400–450 for the posterior LA wall and right atrium [RA]) [15]. During ablation, intraprocedural responses were categorized as follows: (1) VRs, defined as *a* > 20% decrease in heart rate (HR) during sinus rhythm, transient ventricular asystole, or atrioventricular block, serve as optimal electrophysiological markers for GP localization [6, 12, 16]; (2) HR acceleration during ablation [16]; or (3) no observable response.

In the EAGC group, ablation targets were selected based on empirically defined GP sites, as previously described. According to the GPs nomenclature established by Armour et al. [17], the commonly targeted GPs include the RSGP, RIGP, LSGP, LIGP, marshal tract GP (MTGP), posteromedial LA GP (PMLGP), and superior vena cava–aorta GP (SVC‐Ao GP), with their anatomic locations illustrated in the 3D electroanatomic map (Figure 1). The ablation sequence proceeded through RSGP‐LSGP‐MTGP‐LIGP‐ RIGP‐(SVC‐Ao GP)‐PMLGP in consecutive order. Each ganglionated plexus (GP) underwent point‐by‐point radiofrequency delivery until achieving confluent ablation zones with cloud‐like morphology. The corresponding procedural endpoints were defined as disappearance of VRs for cases with positive responses, cessation of HR acceleration for tachycardic responses, or elimination of local electrograms (peak‐to‐peak bipolar amplitude < 0.1 mV) for non‐responsive sites [6].

In the HAFE group, HAFE potentials were systematically mapped at common GP sites within the left and right atria during sinus rhythm. This was performed using a Thermocool Smart‐Touch catheter on the CARTO 3 electro‐anatomic mapping system, with an initial screening step using filter settings of 16–500 Hz and a sweep speed of 200 mm/s, followed by confirmation using filter settings of 200–500 Hz and a sweep speed of 400 mm/s [18]. For each GP region, mapping was performed at a minimum of 5 sites. These potentials were defined by meeting dual criteria: (1) amplitude ≥ 0.7 mV and (2) morphological complexity with ≥4 distinct deflections [18, 19]. The identified HAFE sites were annotated on the three‐dimensional EAM system, with ablation performed in accordance with the sequence protocol established by the EAGC group. The primary procedural endpoints were defined as: (1) near‐complete elimination (<0.1 mV) of all targeted HAFE regions, and (2) complete abolition of positive VRs during radiofrequency energy delivery [18, 19].

### 2.3 Postoperative Management and Follow‐Up

Patients were prescribed oral rivaroxaban (20 mg once daily) for 1 month, with outpatient clinic visits scheduled at 1, 3, and 6 months, followed by biannual assessments, including physical examinations, 12‐lead electrocardiogram (ECG), transthoracic echocardiography, and 24‐h Holter monitoring. Retrospective telephone follow‐up by trained personnel evaluated syncope recurrence and quality of life (QoL) using the Syncope Functional Status Questionnaire (SFSQ [20]). The primary endpoint was syncope‐free survival during the 24‐month follow‐up, while secondary endpoints encompassed procedural efficiency (skin‐to‐skin duration, radiation exposure [mGy], fluoroscopy time [min]), physiological impact (ΔHR: pre‐ vs. post‐procedural resting HR difference), and QoL improvements (via syncope dysfunction score [SDS]). Peri‐procedural complications within 30 days were compared between groups, including access‐site bleeding requiring intervention, hematoma (≥5 cm), arteriovenous fistula, retroperitoneal hematoma, pneumothorax/hemothorax, pulmonary vein stenosis (>50%), LA‐esophageal fistula, cardiac tamponade, and thromboembolic events.

### 2.4 Statistical Analysis

All statistical analyses were conducted using IBM SPSS Statistics (version 27; SPSS Inc, USA) and R version 4.3.0 (R Foundation for Statistical Computing), and all figures were created with GraphPad Prism (version 8.0; GraphPad Software, USA). The threshold for statistical significance was set at a two‐tailed α level of 0.05. Data normality was assessed through the Shapiro−Wilk test. Continuous variables with normal distribution were expressed as mean ± standard deviation (SD), while non‐normally distributed variables were summarized as median (interquartile range, IQR). Categorical variables were presented as absolute numbers with corresponding percentages.

Continuous variables were compared using independent Student's *t*‐tests (parametric data) or Mann−Whitney *U* tests (non‐parametric data). Categorical variables were analyzed with Pearson's *χ*² tests, with Fisher's exact tests applied for cells containing expected frequencies < 5.

Syncope‐free survival was assessed via Kaplan‐Meier curves, with between‐group differences evaluated using log‐rank tests. Cox proportional hazards regression models generated hazard ratios (HR) and 95% confidence intervals (CI) for clinical outcomes.

## 3 Results

### 3.1 Baseline Characteristics

A total of 109 patients were included for analysis (mean age of 57.47 ± 13.77 years), comprising 70 patients in the EAGC group and 39 patients in the HAFE group. Baseline clinical characteristics and comparisons between the EAGC and the HAFE groups are summarized in Table 1. The median number of syncope episodes before catheter ablation (CNA) was comparable between the two groups (Overall: 6 [IQR 4–10]; HAFE: 6 [IQR 4–14] vs. EAGC: 6 [IQR 5–10]; *p* = 0.602).

**Table 1:** Baseline characteristics.

Parameter	Total (*n* = 109)	EAGC (*n* = 70)	HAFE (*n* = 39)	*p*
*Clinical characteristics*				
Age, years	57.47 ± 13.77	58.36 ± 13.75	55.87 ± 13.83	0.369
Male, *n* (%)	60 (55.05)	35 (50.00)	25 (64.10)	0.156
BMI, kg/m^2^	22.91 (20.76, 25.01)	22.94 (20.76, 25.00)	22.83 (21.11, 25.44)	0.822
Syncope episodes before CNA	6.00 (4.00, 10.00)	6.00 (5.00, 10.00)	6.00 (4.00, 14.00)	0.602
Type of syncope, *n* (%)				0.876
Cardioinhibitory	14 (12.84)	9 (12.86)	5 (12.82)	
Vasodepressor	17 (15.60)	12 (17.14)	5 (12.82)	
Mixed	61 (55.96)	39 (55.71)	22 (56.41)	
Missing/unclassified	17 (15.60)	10 (14.29)	7 (17.95)	
LVEF, %	66.79 ± 4.81	66.39 ± 4.89	67.51 ± 4.64	0.243
LA, mm	33.41 ± 4.52	33.14 ± 4.54	33.90 ± 4.49	0.406
LVEDD, mm	46.04 ± 4.31	45.90 ± 4.15	46.28 ± 4.64	0.660
LVESD, mm	28.93 ± 3.18	29.03 ± 3.06	28.74 ± 3.42	0.656
*Medical history*				
Hypertension, *n* (%)	42 (38.53)	28 (40.00)	14 (35.90)	0.673
T2DM, *n* (%)	9 (8.26)	5 (7.14)	4 (10.26)	0.839
CHD, *n* (%)	3 (2.75)	1 (1.43)	2 (5.13)	0.602
Carotid atherosclerosis, *n* (%)	5 (4.59)	4 (5.71)	1 (2.56)	0.783
AF, *n* (%)	8 (7.34)	5 (7.14)	3 (7.69)	1.000

*Note: p* value for syncope subtype comparison (cardioinhibitory, vasodepressor, mixed) was based on 92 patients with known subtypes, excluding missing data.

Abbreviations: AF, atrial fibrillation; BMI, body mass index; CHD, coronary heart disease; CNA, cardioneuroablation; EAGC, empirical anatomical‐guided cardioneuroablation; HAFE, high‐amplitude fractionated electrogram‐guided Cardioneuroablation; LA, left atrial diameter; LVEDD, left ventricular end‐diastolic diameter; LVEF, left ventricular ejection fraction; LVESD, left ventricular end‐systolic diameter; T2DM, type 2 diabetes mellitus.

The groups were well‐balanced at baseline, with no significant differences in demographic profiles (age, sex, and BMI), cardiac structure and function (ejection fraction, LA and ventricular dimensions), comorbidities (hypertension, diabetes, coronary disease, carotid atherosclerosis, atrial fibrillation), or distributions of VVS subtypes (cardioinhibitory, vasodepressor, mixed) (all *p* > 0.05).

No statistically significant differences were observed in the HUTT parameters between the two groups (Supporting Information S2: Table 1).

### 3.2 Primary Endpoint Assessment

**Figure 2:**
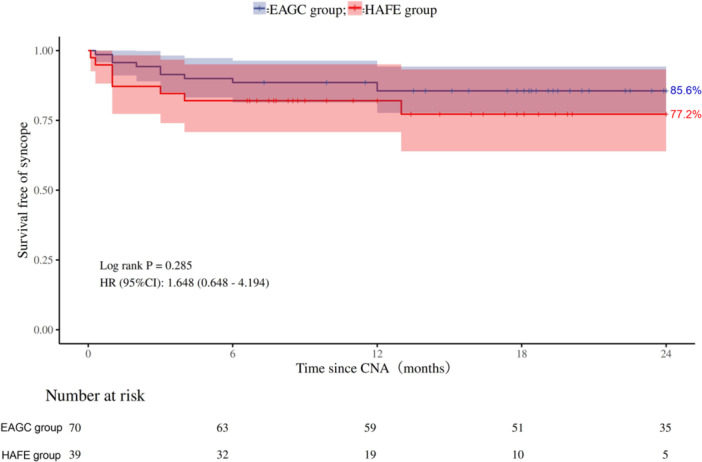
Kaplan−Meier analysis of 24‐month syncope‐free survival comparing HAFE‐guided CNA versus EAGC. The 24‐month syncope‐free survival rate was 85.6% in the EAGC group and 77.2% in the HAFE group. Abbreviations: EAGC group, empirical anatomical‐guided cardioneuroablation group; HAFE group, high‐amplitude fractionated electrogram‐guided cardioneuroablation group.

The 24‐month syncope‐free survival rate was 85.6% in the EAGC group and 77.2% in the HAFE group. Kaplan−Meier survival analysis assessing 24‐month syncope‐free rates revealed no statistically significant differences between the treatment groups (log‐rank test, *p* = 0.285; Figure 2). Comparative analysis demonstrated a non‐significant increase in syncope recurrence risk for the HAFE group relative to the EAGC group, with a HR of 1.648 (95% CI 0.648−4.194). Multivariable Cox regression analysis with syncope as the endpoint showed that, after adjusting for potential confounders including sex and type of syncope, the risk of syncope recurrence was not significantly different between the HAFE and EAGC groups (HR = 1.05, 95% CI 0.36–3.09, *p* = 0.932; Supporting Information S1: Table 2).

### 3.3 Secondary Endpoint Assessment

**Figure 3:**
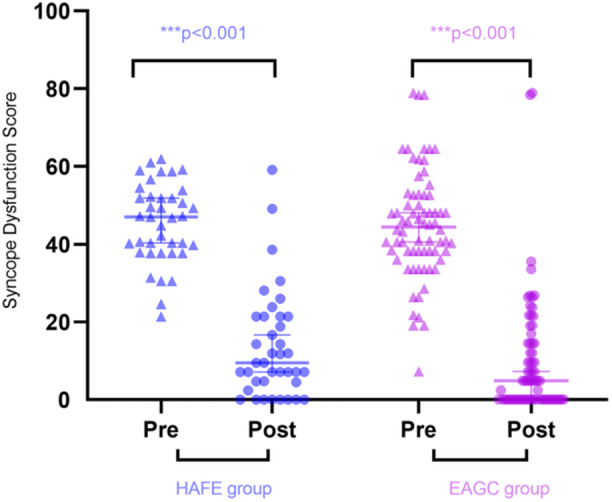
Pre‐ versus post‐intervention quality of life changes in HAFE and EAGC groups. Abbreviations as in Figure 2.

Secondary outcome measures are comprehensively presented in Table 2. Postprocedural HR elevation showed no statistically significant intergroup variation (ΔHR: 20.00 [IQR 12.00–27.75] vs. 15.00 [IQR 9.00–25.00] bpm; *p* = 0.069). The HAFE group demonstrated shorter procedural duration [median 66.00 min (IQR 52.00–87.50) versus 90.00 min (71.75–101.50) in the EAGC group; *p* < 0.001] and reduced radiation exposure (X‐ray dose: 6.00 mGy [4.17–10.75] vs. 12.00 mGy [6.48–20.00], *p* = 0.002; fluoroscopy time: 2.53 min [1.92–4.06] versus 4.38 min [2.47–7.05], *p* < 0.001) compared to the EAGC group. Notably, both treatment arms exhibited significant within‐group improvements in SDS (both *p* < 0.001). However, the intergroup comparison revealed no statistically significant difference in score reduction magnitude (ΔSDS difference *p* = 0.487; Figure 3).

**Table 2:** Procedural outcomes of EAGC versus HAFE‐guided CNA.

Parameter	Total (*n* = 109)	EAGC (*n* = 70)	HAFE (*n* = 39)	*p*
ΔHR, bpm	19.00 (10.00, 27.00)	20.00 (12.00, 27.75)	15.00 (9.00, 25.00)	0.069
Procedure Time, min	83.00 (65.00, 98.00)	90.00 (71.75, 101.50)	66.00 (52.00, 87.50)	< 0.001
Radiation exposure, mGy	9.85 (5.00, 18.00)	12.00 (6.48, 20.00)	6.00 (4.17, 10.75)	0.002
Fluoroscopy Time, min	3.40 (2.29, 5.38)	4.38 (2.47, 7.05)	2.53 (1.92, 4.06)	< 0.001

Abbreviations: ΔHR, change in heart rate between postoperative and preoperative measurements; other abbreviations as in Table 1.

### 3.4 Safety Endpoint Assessment

Both groups exhibited comparable procedural safety profiles. Cardiac tamponade occurred intraprocedurally in one patient from the EAGC group (1/70, 1.43%) and one patient from the HAFE group (1/39, 2.56%). Both cases were effectively managed through immediate percutaneous pericardiocentesis combined with catheter drainage, achieving complete hemodynamic stabilization. Furthermore, comprehensive perioperative monitoring revealed no instances of procedure‐ or device‐related complications in either group, including thromboembolic events (e.g., stroke or systemic embolism), vascular access injuries (e.g., hematoma or pseudoaneurysm), or all‐cause mortality.

## 4 Discussion

### 4.1 Main Findings

Our study revealed the following key findings: (1) Long‐term efficacy analysis showed no statistically significant difference between EAGC and HAFE‐guided CNA, with comparable 24‐month syncope‐free survival rates and recurrence risk; (2) The EAGC group demonstrated more extensive GP ablations, longer intervention times, and higher radiation exposure than those in the HAFE group, yet both approaches achieved similar functional improvements in QoL; (3) Safety profiles were comparable for both strategies.

### 4.2 EAGC

The empirical anatomical‐ guided CNA is a fundamental, safe, and effective technique [5, 6, 7, 8, 21]. It involves empirical ablation at presumed anatomical locations of GP. While its procedural simplicity is an advantage, variations exist between centers regarding the cardiac chambers ablated, GP targets, and ablation sequence due to anatomical variations in GP distribution and the lack of a standardized protocol. For example, Sun et al. [21] employed a LA ablation sequence for VVS as follows: LSGP‐MTGP‐LIGP‐RSGP‐RIGP. Qin et al. [6] performed bilateral atrial ablation in the sequence: LSGP‐LIGP‐RSGP‐RIGP‐(SVC‐Ao GP). Hu et al. [22] compared VR, HR, and blood pressure (BP) characteristics during LA CNA using two sequences (Group A: LSGP‐LIGP‐RIGP‐RAGP; Group B: RAGP‐LSGP‐LIGP‐RIGP). Their study found that the sequence of GP ablation influenced the incidence of VR and BP reduction during CNA, identifying RAGP as a key target for increasing HR and suppressing decreases in VR and BP intraprocedurally. In our study, we targeted seven common GP regions in the following sequence: RSGP‐LSGP‐MTGP‐LIGP‐RIGP‐(SVC‐Ao GP)‐PMLGP. While potentially prolonging procedure time, this approach aims to ablate as many GPs as possible, thereby mitigating the impact of varying numbers of GP ablations across studies on outcomes. Consistent with Hu et al.'s findings, [5, 22] our study also observed that VR occurred most frequently during LSGP ablation (50 cases, 41.67%), while RSGP ablation often elicited HR acceleration (15 cases, 12.5%). We commenced ablation at the RSGP first, as it was a critical target for increasing HR and inhibiting VR and BP reduction during the procedure.

### 4.3 Electrogram‐Guided CNA

The distal atrial insertion sites of GP exhibit highly fractionated atrial electrograms (EGMs), distinct from the surrounding non‐fractionated or minimally fractionated myocardial EGMs, providing a theoretical basis for GP identification via EGM characteristics [12]. This is explained by the neuromyocardial interface, where scattered atrial myocytes intermingle with intrinsic cardiac neurons, producing fractionated electrograms (AF‐Nests) that colocalize with GPs [23]. Pachon et al. [1] utilized spectral analysis for GP localization, though this method requires specialized software. Lellouche et al. [10], building on Pachon's AFN concept, classified atrial electrograms into three types: normal (<4 deflections), low‐amplitude fractionated (amplitude < 0.7 mV, ≥4 deflections), and HAFE (amplitude ≥ 0.7 mV, ≥4 deflections), and found HAFE associated with VRs during ablation at suspected GP sites. Aksu et al. [18, 19] simplified this method by utilizing the filtering capabilities of standard electrophysiology equipment (a key advantage), setting the band‐pass filter between 200 and 500 Hz to identify HAFE in conventional GP regions. Although HAFE‐guided CNA has demonstrated safety and efficacy in studies [11, 24, 25], its specificity and sensitivity have limitations: atrial fibrosis or pulmonary vein interference may obscure fractionated potentials, and clinically effective GP ablation sites sometimes lack detectable HAFE signals. Furthermore, no consensus exists on the recommended catheter type for HAFE mapping (e.g., decapolar circular catheter [23], ablation catheter, or mapping catheter [26]). In this study, HAFE mapping during sinus rhythm—using a Thermocool Smart‐Touch catheter on the CARTO 3 electro‐anatomic mapping system with an initial 16–500 Hz filter (200 mm/s) followed by a confirmatory 200–500 Hz filter (400 mm/s)—proved to be a feasible and effective approach. Nevertheless, we acknowledge that mapping catheters with narrower interelectrode spacing (e.g., 1–2 mm) may further enhance the detection sensitivity of HAFE potentials. The median number of deflections in detected HAFEs was 8 (IQR: 7–10), with a median amplitude of 1.40 mV (IQR: 1.05–1.94 mV). HAFEs were most frequently observed at RSGP (38 cases, 95.0%) and LSGP (34 cases, 85.0%), followed by LIGP (28 cases, 70.0%), SVC‐Ao GP (26 cases, 65.0%), and RIGP (24 cases, 60.0%); occurrence was lower at MTGP (12 cases, 30.0%) and PMLGP (6 cases, 15.0%). This finding suggests RSGP and LSGP may be critical sites for CNA.

### 4.4 Comparison of CNA Strategies

Current intraprocedural methods for identifying ablation targets include: HFS, anatomical landmark‐based localization, and endocardial EGM‐guided strategies [12]. However, randomized controlled trial (RCT) evidence evaluating the clinical efficacy of these methods remains scarce. Existing meta‐analyses report conflicting conclusions: Vandenberk B et al. [27] found no significant difference in syncope recurrence rates between different GP targeting techniques, while Prata AA et al. [28] observed a greater prevalence of syncope (12%, 95% CI 7%–17%) and post‐procedure prodromal symptoms with the isolated Anatomical Approach. Aksu et al. [18] conducted a cross‐sectional observational study comparing EAM‐guided CNA with a combined approach (EAM + HFS) for VVS, showing comparable median event‐free survival between groups (*χ*² = 0.03, *p* = 0.87). Our retrospective case‐control study demonstrated comparable efficacy between EAGC and HAFE‐guided CNA in reducing syncope recurrence in patients with refractory VVS. Consistent with Aksu et al.'s findings, [18, 29] this study also found that HAFE‐guided CNA significantly reduced procedure time and radiation exposure compared to EAGC. Specifically, the shorter fluoroscopy time in the HAFE group may be explained by the number of ablation sites. In both groups, fluoroscopy was occasionally used to confirm catheter position. Because HAFE potentials were not present at all predefined GP sites, ablation in the HAFE group was performed at fewer sites than in the EAGC group, where all predetermined locations were ablated. As total fluoroscopy time tends to increase with more ablation sites, the smaller ablation scope in the HAFE group likely contributed to the observed reduction. Nevertheless, we acknowledge that this interpretation remains hypothesis‑generating, as our retrospective design did not capture step‑by‑step fluoroscopy usage. Therefore, this experience requires confirmation through larger prospective, multicentre RCTs with detailed procedural time‑motion analysis.

### 4.5 Functional Improvement and QoL

Although VVS typically follows a benign course, it carries multidimensional risks, including trauma from sudden syncope, diagnostic challenges due to symptom overlap with life‐threatening cardiovascular conditions, and hazards in specific situations (e.g., near water or while driving). Importantly, recurrent VVS significantly impairs QoL through persistent anxiety, reduced physical/social activities, and occupational limitations, creating a vicious cycle of psychological distress and functional impairment [13]. Clinical evidence indicates that CNA not only reduces syncope recurrence but also significantly improves patient‐reported QoL outcomes [30, 31]. This study showed significant reductions in SDS from baseline to follow‐up in both the EAGC and HAFE groups, highlighting the dual benefit of CNA in improving both clinical events and mental well‐being. The similar degree of SDS improvement between groups suggests functional recovery may be independent of the specific ablation method. However, limited by the sample size of this single‐center retrospective study, larger future studies are needed to validate potential differences in SDS scores between techniques. These findings support the integration of QoL metrics, such as the SDS, into standardized efficacy assessments for VVS treatment regimens, complementing traditional clinical outcome parameters.

## 5 Limitations

This study has several limitations: (1) Although this was a single‐center, non‐randomized retrospective analysis, we implemented rigorous inclusion and exclusion criteria, achieved balanced baseline characteristics between groups, and performed multivariate adjustment for potential confounders to minimize bias as much as possible. Nevertheless, future multicenter prospective RCTs are warranted to further validate our findings. (2) Given the episodic nature of VVS, while the 24‐month follow‐up period is scientifically reasonable, longer‐term follow‐up is warranted to fully evaluate the procedural efficacy of CNA. (3) Methodological limitations include the use of an ablation catheter for HAFE mapping; its relatively wide interelectrode spacing may have compromised detection sensitivity. Future investigations using dedicated narrow‐spacing mapping catheters may achieve higher sensitivity and specificity. Additionally, the exclusive use of the CARTO 3 EAM system (Biosense Webster) represents another limitation. The generalizability of the results to other platforms (e.g., EnSite Precision system, Abbott) and different energy modalities (e.g., pulsed‐field ablation [32] or balloon cryoablation [33]) requires further validation through multicentre comparative studies. Furthermore, this study lacked extracardiac vagal stimulation (ECVS) to assess the completeness of denervation during and at the end of the procedure. The RSGP‐first ablation sequence was used in this study. While improving intraoperative safety, this sequence may have masked VRs during subsequent GP ablation. Future prospective studies are needed to compare the effects of different ablation sequences on efficacy and safety, and to determine the optimal order.

## 6 Conclusions

This comparative study of EAGC and HAFE‐guided CNA for refractory VVS revealed comparable long‐term efficacy and safety between the two approaches. HAFE‐guided CNA offered procedural advantages (reduced operative duration and radiation exposure) while maintaining comparable outcomes in this cohort, warranting confirmation in prospective multicenter studies.

## Author Contributions

Bo Zhang performed the experiments, analyzed the data, and wrote the first draft of the manuscript. Xianfeng Du conceived and designed the study, supervised the project, and revised the manuscript. The other authors (Chenxu Luo, Xinzhi Yu, Shenyuan Zhou, Yongxing Jiang, Yin Xu, Renyuan Fang, Mingjun Feng, and Caijie Shen) contributed to data collection and analysis, and provided critical feedback on the manuscript. All authors read and approved the final manuscript.

## Disclosure

The authors have nothing to report.

## Conflicts of Interest

The authors declare no conflicts of interest.

## Supporting information

Supporting File 1

Supporting File 2

## Data Availability

The data that support the findings of this study are available from the corresponding author upon reasonable request. The datasets used and/or analyzed during the current study are available from the corresponding author on reasonable request.
