# Supplementary material for: Empirical Anatomical Versus HAFE‐Guided Cardioneuroablation for Vasovagal Syncope: Long‐Term Efficacy and Safety Outcomes
Source: Clin Cardiol. 2026 May 18;49(5):e70348. doi: 10.1002/clc.70348 (PMC13181592; doi:10.1002/clc.70348)
Supplement: Supplementary file 1 — Supporting File 1 [file CLC-49-e70348-s001.docx]

**Contents of Supplemental Appendix**

Supplemental Figure 1: Study flowchart ................................................. S2

Supplemental Table 1: Head-up Tilt Test Data ..........................................S3

**Supplemental Figure 1: Study flowchart.**


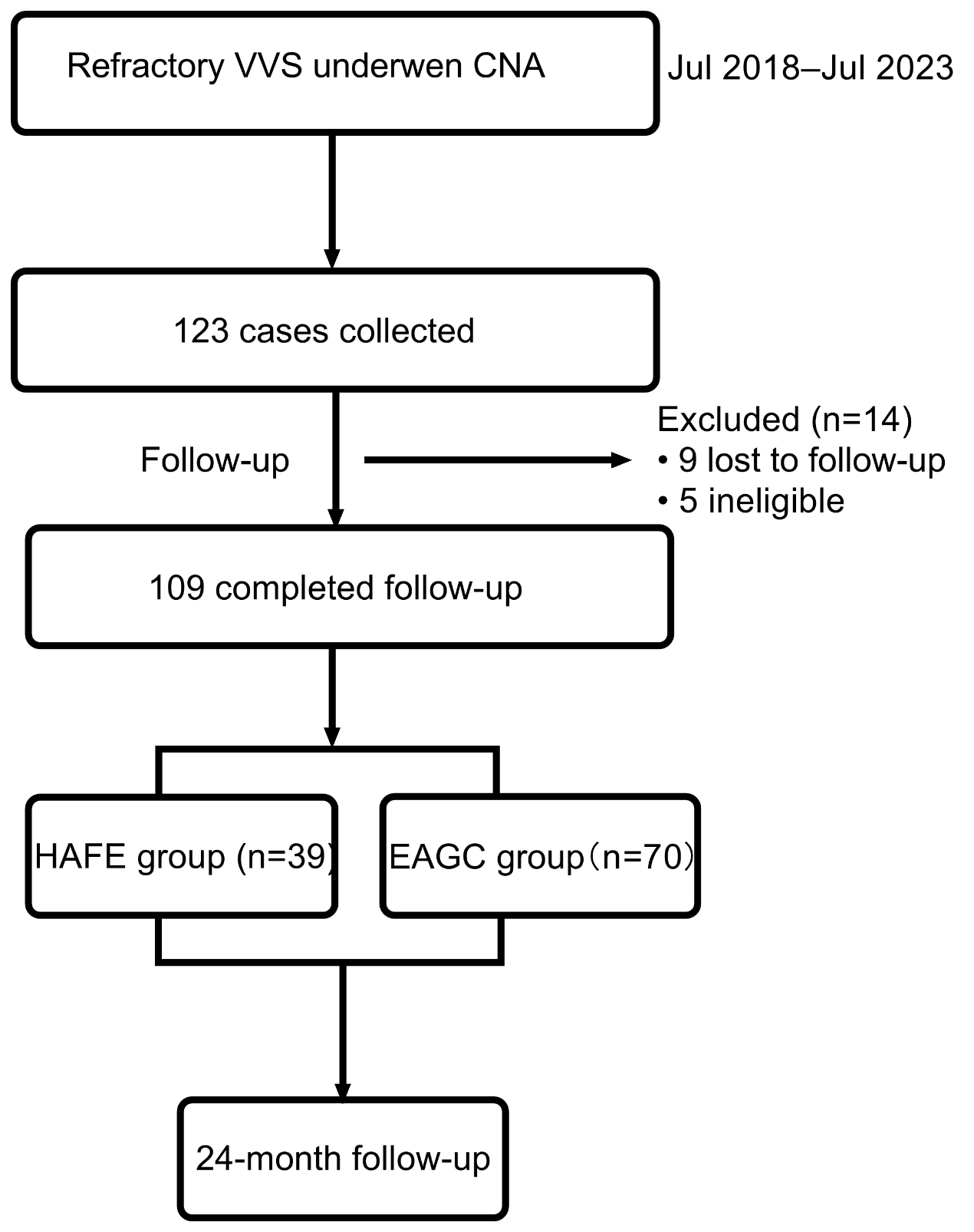


Abbreviations: VVS, vasovagal syncope; CNA, cardioneuroablation; EAGC group, empirical anatomical-guided cardioneuroablation group;

HAFE group, high-amplitude fractionated electrogram-guided cardioneuroablation group

**Supplemental Table 1. Head-up Tilt Test Data**

| Parameter | Total (n = 109) | EAGC (n = 70) | HAFE (n = 39) | *P* |
| --- | --- | --- | --- | --- |
|  |  |  |  |  |
| Supine HR, bpm | 68.00 (60.00, 76.00) | 65.50 (60.00, 76.00) | 68.00 (58.00, 78.00) | 0.615 |
| Supine SBP, mmHg | 122.00 (114.00, 144.00) | 123.50 (114.00, 140.25) | 121.00 (114.00, 146.00) | 0.694 |
| Supine DBP, mmHg | 75.00 (70.00, 81.00) | 75.00 (69.00, 81.00) | 75.00 (70.00, 81.00) | 0.448 |
| HR at End of Baseline Phase, bpm | 75.00 (69.00, 89.00) | 75.00 (69.00, 86.00) | 78.00 (68.00, 94.00) | 0.489 |
| SBP at End of Baseline Phase, mmHg | 119.00 (111.00, 126.00) | 118.50 (110.50, 126.00) | 121.00 (111.00, 129.00) | 0.710 |
| DBP at End of Baseline Phase, mmHg | 78.00 (73.00, 84.00) | 78.00 (71.75, 84.00) | 79.00 (74.00, 85.00) | 0.351 |
| Peak HR during medication challenge, bpm | 95.90 ± 20.83 | 94.91 ± 21.74 | 97.82 ± 19.19 | 0.551 |
| Peak SBP during medication challenge, mmHg | 112.88 ± 16.62 | 111.07 ± 16.68 | 116.36 ± 16.23 | 0.174 |
| Peak DBP during medication challenge, mmHg | 75.00 ± 10.82 | 74.54 ± 11.00 | 75.89 ± 10.61 | 0.594 |
| Time from medication challenge to Syncope, min | 5.00 (4.00, 8.00) | 5.00 (4.00, 8.00) | 5.50 (4.00, 8.00) | 0.337 |
| SBP Decrease, mmHg | 77.50 (41.50, 119.25) | 89.00 (44.00, 117.00) | 61.00 (35.00, 128.00) | 0.393 |
| DBP Decrease, mmHg | 43.00 (29.00, 76.00) | 58.00 (31.00, 76.00) | 40.00 (27.00, 80.00) | 0.837 |
| HR Decrease, bpm | 21.00 (8.00, 37.50) | 21.00 (10.50, 44.50) | 21.50 (6.50, 29.25) | 0.466 |

Abbreviations: HR, heart rate (beats per minute, bpm); SBP/DBP, systolic/diastolic blood pressure (mmHg); min, minutes; pharmacologic provocation, drug-induced challenge test.
