# Supplementary material for: Empirical Anatomical Versus HAFE‐Guided Cardioneuroablation for Vasovagal Syncope: Long‐Term Efficacy and Safety Outcomes
Source: Clin Cardiol. 2026 May 18;49(5):e70348. doi: 10.1002/clc.70348 (PMC13181592; doi:10.1002/clc.70348)
Supplement: Supplementary file 2 — Supporting File 2 [file CLC-49-e70348-s002.docx]

| Variables | Univariate | | | | | Multivariate | | | | |
| --- | --- | --- | --- | --- | --- | --- | --- | --- | --- | --- |
|  | β | S.E | Z | *P* | HR (95%CI) | β | S.E | Z | *P* | HR (95%CI) |
| EAGC |  |  |  |  | 1.00 (Reference) |  |  |  |  | 1.00 (Reference) |
| HAFE | 0.50 | 0.48 | 1.05 | 0.294 | 1.65 (0.65 ~ 4.19) | 0.05 | 0.55 | 0.09 | 0.932 | 1.05 (0.36 ~ 3.09) |
| Male |  |  |  |  | 1.00 (Reference) |  |  |  |  | 1.00 (Reference) |
| Female | -0.07 | 0.47 | -0.15 | 0.879 | 0.93 (0.37 ~ 2.36) | -0.08 | 0.52 | -0.15 | 0.884 | 0.93 (0.33 ~ 2.58) |
| Type of syncope |  |  |  |  |  |  |  |  |  |  |
| Cardioinhibitory |  |  |  |  | 1.00 (Reference) |  |  |  |  | 1.00 (Reference) |
| Vasodepressor | -0.16 | 0.82 | -0.20 | 0.842 | 0.85 (0.17 ~ 4.21) | -0.17 | 0.82 | -0.21 | 0.835 | 0.84 (0.17 ~ 4.22) |
| Mixed | -0.38 | 0.67 | -0.58 | 0.564 | 0.68 (0.18 ~ 2.51) | -0.38 | 0.67 | -0.58 | 0.565 | 0.68 (0.18 ~ 2.52) |

**Supplemental Table 2. Univariate and Multivariate Cox Regression Analysis for Syncope Recurrence**

Abbreviations: EAGC, empirical anatomical-guided cardioneuroablation group;

HAFE, high-amplitude fractionated electrogram-guided cardioneuroablation group

HR: Hazard Ratio, CI: Confidence Interval
